# Sensor-Based Auto-Focusing System Using Multi-Scale Feature Extraction and Phase Correlation Matching

**DOI:** 10.3390/s150305747

**Published:** 2015-03-10

**Authors:** Jinbeum Jang, Yoonjong Yoo, Jongheon Kim, Joonki Paik

**Affiliations:** 1 Image Processing and Intelligent System Laboratory Graduate School of Advanced Imaging Science, and Film Chung-Ang University, Seoul 156-756, Korea; E-Mails: jinbeum23@cau.ac.kr (J.J.); whitener@cau.ac.kr (Y.Y.); 2 Digital Design Team, SK Hynix, Gyeonggi-do 463-844, Korea; E-Mail: jongheon.kim@sk.com

**Keywords:** auto-focusing, phase-difference, black mask, difference of Gaussian, phase correlation, hybrid auto-focusing

## Abstract

This paper presents a novel auto-focusing system based on a CMOS sensor containing pixels with different phases. Robust extraction of features in a severely defocused image is the fundamental problem of a phase-difference auto-focusing system. In order to solve this problem, a multi-resolution feature extraction algorithm is proposed. Given the extracted features, the proposed auto-focusing system can provide the ideal focusing position using phase correlation matching. The proposed auto-focusing (AF) algorithm consists of four steps: (i) acquisition of left and right images using AF points in the region-of-interest; (ii) feature extraction in the left image under low illumination and out-of-focus blur; (iii) the generation of two feature images using the phase difference between the left and right images; and (iv) estimation of the phase shifting vector using phase correlation matching. Since the proposed system accurately estimates the phase difference in the out-of-focus blurred image under low illumination, it can provide faster, more robust auto focusing than existing systems.

## 1. Introduction

Auto-focusing (AF) is one of the most fundamental techniques for acquiring high-quality images using a digital camera and has evolved for the past few decades [1]. Various AF techniques can be classified into active and passive approaches. In order to measure the distance between an object and camera, the former uses an additional signal, such as ultra-sound or a laser beam [2,3], whereas the latter analyzes the incident light beam passing through the optical system [4,5]. Passive auto-focusing techniques are further classified into contrast detection auto-focusing (CDAF) and phase detection auto-focusing (PDAF) approaches. Most consumer digital cameras adopt either a CDAF or PDAF auto-focusing system. In order to take advantages of both active and passive AF systems, a hybrid AF technique has recently attracted increasing attention. More specifically, the hybrid method roughly moves the focusing lens to the near optimal focusing position using either an active or PDAF technique and then accurately moves the focusing lens using CDAF [6].

In the hybrid AF system, the first focusing step should quickly move the focusing lens as close to the optimal focusing position as possible for the second focusing step to minimize traveling back and forth around the peak of the contrast. Thus, a manual focusing technique uses a specially-designed imaging sensor to compute the phase difference. In this system, two AF points are differently masked to take different phases. However, the mask reduces the amount of incoming light, and it is not easy to extract features in the input defocused images. In order to solve these problems, a robust feature extraction from the defocused image and the robust phase detection methods are needed.

The proposed system performs multi-scale feature extraction from severely defocused input images and estimates the phase difference using phase correlation. The paper is organized as follows: Section 2 presents an in-depth summary of existing AF techniques, and Section 3 presents the proposed sensor-based robust AF system. Experimental results are presented in Section 4, and Section 5 concludes the paper.

## 2. Theoretical Background

### 2.1. Phase-Difference Detection Auto-Focusing

Phase-difference detection auto-focusing (PDAF) is a passive method that adjusts focus using the phase difference from a pair of linear focusing sensors. Most of digital single-lens reflex (DSLR) cameras are equipped with the PDAF system, where the AF lens moves to the in-focus position faster than other types of auto-focusing systems [7,8].

**Figure 1.**
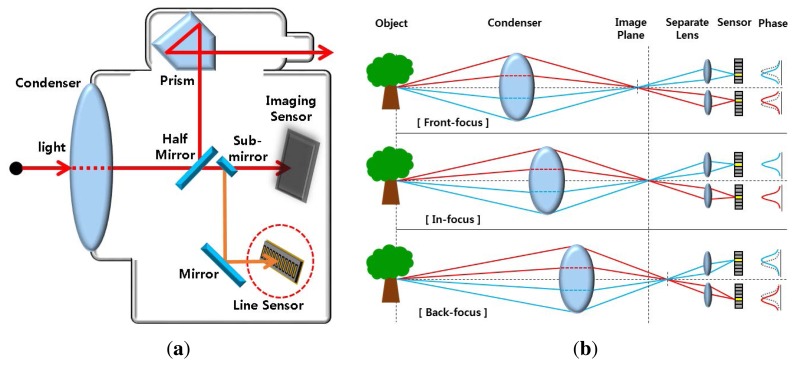
Phase-difference auto-focusing (PDAF) system: **(a)** light path in the camera with the PDAF system and **(b)** the concept of phase difference generated by two line sensors.

Figure 1a shows a fundamental PDAF model, where a part of the incoming light to the camera is reflected by the half sub-mirror down to the line sensor. The light reflected down for focusing converges onto two separate line sensors by the separate lens, as shown in Figure 1b [9]. If an object is located at the in-focus position, its image is acquired in the same position of the two line sensors. On the other hand, if an object is located at the near-focus position, two images comes closer, and *vice versa*. As a result, the focusing lens moves according to the distance between two images.

The PDAF system can quickly find the in-focus position of the lens using additional line sensors that increase the hardware cost. Another drawback of the PDAF system is that the focusing accuracy is highly dependent on the geometric structure of the half mirror and separating lens [10].

### 2.2. Contrast Detection Auto-Focusing

The contrast detection auto-focusing (CDAF) system detects the optimal position of the focusing lens by analyzing the high-frequency energy in the acquired image [11]. The CDAF method uses an image processing algorithm that estimates the strength of the edge in the acquired image, and the focusing lens moves back and forth until the high-frequency energy becomes the maximum [12]. In estimating the high-frequency energy, various approaches were proposed using the discrete cosine transform [11,13], gradient [14,15] and Bayesian spectral entropy [16].

The CDAF system is very efficient to implement, since it uses only digital image processing algorithm without additional devices, such as a half mirror or separating lens. However, it takes a longer time to search the optimal lens position, and it is also very sensitive to noise [17].

### 2.3. Auto-Focusing Using a Time-of-Flight Sensor

The time-of-flight auto-focusing (ToFAF) system actively radiates a special light traveling back to the sensor after being reflecting by an object and then measures the time of the travel to estimate the distance of the object from the camera [18–20]. Using the relationship between the distance of the object and the correspondingly optimal in-focus lens position, the ToFAF system can predict the best lens position.

Since the ToFAF method directly computes the distance of an object, it can provide accurate auto-focusing for both low light and flat objects without a sufficient amount of detail. However, the finite range of focusing distance is a fundamental limitation of the ToFAF system, and the pair of an additional light source and focusing sensor is another drawback of the ToFAF system in the sense of efficient implementation.

### 2.4. Sensor-Based Phase-Difference Detection Auto-Focusing

**Figure 2.**
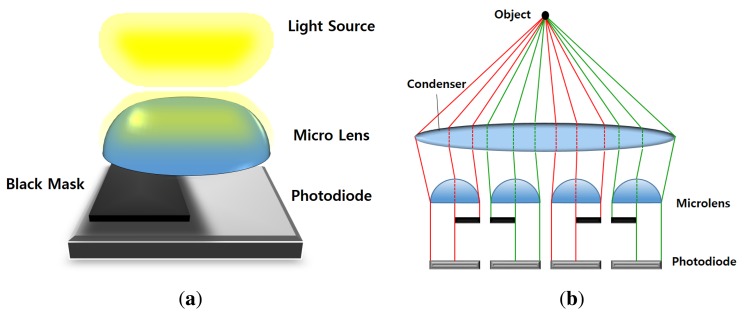
The sensor-based PDAF system: **(a)** the photodiode sensor model; and **(b)** the concept of generating the phase difference in the sensor-based AF system.

In order to remove the additional optical parts and focusing sensors, the sensor-based PDAF system utilizes the imaging sensor for detecting the phase difference [21]. Figure 2 shows the photodiode sensor model of a sensor-based PDAF system and the concept of generating the phase difference in the system [22,23]. As shown in Figure 2a, the black mask is deployed for generating the phase difference. More specifically, each AF point pair is installed in an adjacent place, as shown in Figure 2b. Given the phase difference using the sensor-based PDAF system, the rest of the AF process is performed in the same manner as the PDAF system. Sensor-based PDAF does not require additional hardware or optical parts, such as PDAF, and is as fast as PDAF, since it shares the common concept of phase difference. Because of the black mask, however, low light images are obtained with the additive random noise in this system.

## 3. Sensor-Based PDAF System

The proposed sensor-based AF system consists of three functional modules: (i) acquisition of phase signals or images; (ii) robust feature extraction; and (iii) feature-based motion estimation of two images. After a brief overview of the proposed system, each module is described in the following subsections.

### 3.1. Overview of the Proposed Sensor-Based PDAF System

**Figure 3.**
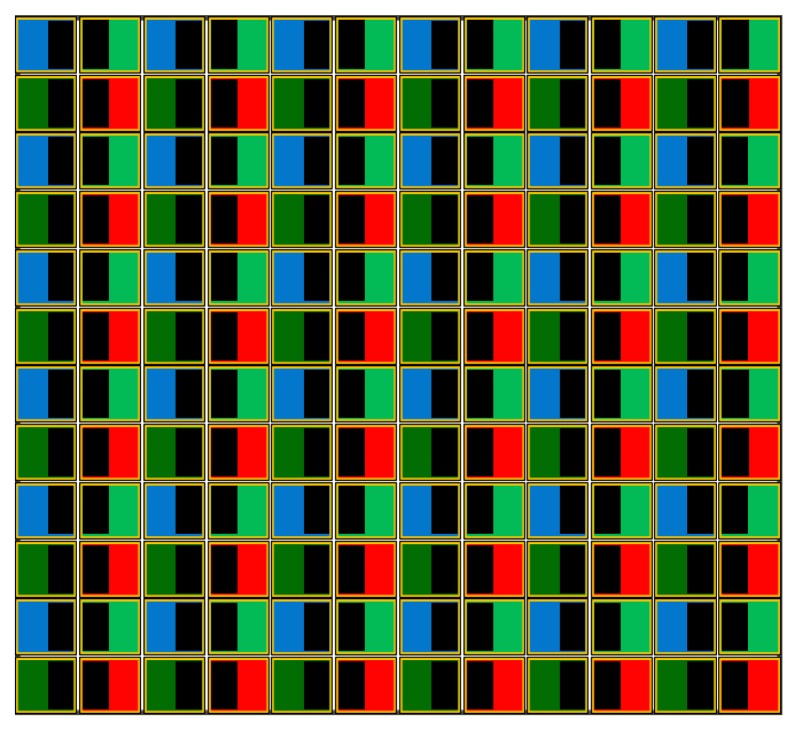
The phase detection sensor for the proposed sensor-based PDAF system. Green masks in the odd and even lines have different brightness, since a real system has different sensitivities.

The proposed AF system requires a special imaging sensor, called the phase detection sensor, as shown in Figure 3, under three assumptions: (i) the AF points in the imaging sensor are placed in all pixels; (ii) each AF point is covered by a micro-lens; and (iii) the BGGRstructure is used for the Bayer color filter array (CFA) [24]. As shown in Figure 3, the right half areas of odd pixels in the horizontal direction are covered by a mask, while the left areas of even pixels are covered by masks. As a result, the proposed system can acquire the phase difference images from AF points in every pixel position.

The image formation model of the proposed PDAF sensor is defined as:

(1) $$\begin{array}{l} g\left(x,y\right)=g_{L}\left(x,y\right)+g_{R}\left(x,y\right),\\ \text{where}\;\{\begin{array}{c} g_{L}\left(x,y\right)=f\left(x,y\right)*h_{L}\left(x,y\right)+\eta \left(x,y\right)\\ g_{R}\left(x,y\right)=f\left(x,y\right)*h_{R}\left(x,y\right)+\eta \left(x,y\right) \end{array} \end{array}$$

and *g*(*x*, *y*) represents an input image, *f*(*x*, *y*) represents a virtually ideal image without focus blur in the Bayer pattern, as shown in Figure 3, *g_L_*(*x*, *y*) and *g_R_*(*x*, *y*), respectively, the left- and right-phase images degraded by the defocus blur, *h_L_*(*x*, *y*) and *h_R_*(*x*, *y*), respectively, the point spread functions (PSFs) of *g_L_*(*x*, *y*) and *g_R_*(*x*, *y*), η(*x*, *y*) the sensor noise, (*x*, *y*) the coordinate of the two-dimensional image and “*” the two-dimensional convolution operation.

**Figure 4.**
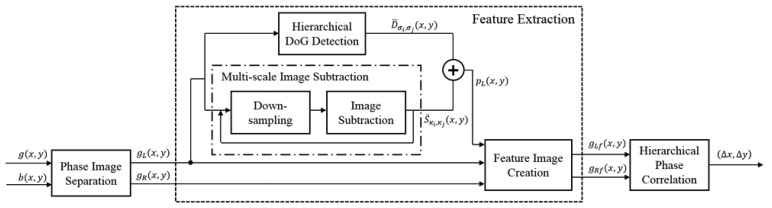
The framework of the proposed system.

Figure 4 shows the entire framework of the proposed AF system. Given the defocused image *g*(*x*, *y*) and the pre-defined AF pattern image *b*(*x*, *y*), left and right phase images are generated in a specific region of interest (ROI) for only green pixels. Next, two cropped images are obtained using the coordinate of the strong feature from the left-phase image, such as the dotted line. In order to extract the strongest feature under a noisy and blurred environment, the difference of Gaussian (DoG) and the difference image of *g_L_*(*x, y*) and *g_R_*(*x*, *y*) are obtained in the proposed system. After down-sampling, as shown in the dash-dotted box in Figure 4, the hierarchical DoG image *D̃*_σ_*i*_, σ_*j*__ (*x*, *y*) and the difference image *S̃*_*κ_i_*, *κ_j_*_ (*x*, *y*) are acquired from the sampled left-phase image *g_L_*(*x*, *y*). *p_L_*(*x*, *y*) is then obtained by adding *D̃*_σ_*i*_, σ_*j*__ (*x*, *y*) and *S̃*_*κ_i_*, *κ_j_*_ (*x*, *y*). In the feature extraction process, the cropped images *g_L_f*(*x*, *y*), and *g_R_f*(*x*, *y*), are obtained from *g_L_*(*x, y*), *g_R_*(*x*, *y*), and *p_L_*(*x*, *y*). Finally, the proposed system obtains the motion vector (Δ*x*, Δ*y*) to compute the phase difference of two images using the hierarchical phase correlation between two phase images.

### 3.2. Acquisition of Phase Images

Assuming that the color filter array (CFA) has the Bayer BGGR pattern, a pair of adjacent sets of AF points is used to generate two phase images. Since only green pixels are used, odd pixels take the light coming into the left side of the photodiode and even pixels take the light coming into the right side. As a result, sampled odd pixels generate the left phase image, and sampled even pixels generate the right phase image.

**Figure 5.**
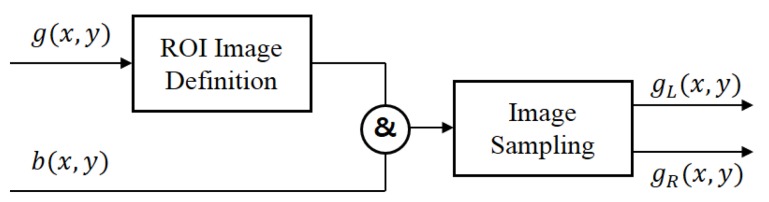
The separating process of phase images from an input image.

Figure 5 shows the phase image separation process, where *b*(*x*, *y*) represents a binary image defining the positions of AF points and *g_L_*(*x*, *y*) and *g_R_*(*x*, *y*) the left and right phase images, respectively. The pre-specified region in *g*(*x*, *y*) is multiplied by the binary image *b*(*x*, *y*) and then sample only green pixels to generate the green intensity images in the ROI. *g_L_*(*x*, *y*) is obtained from the even lines, and *g_R_*(*x*, *y*) is obtained from the odd lines of the ROI image.

### 3.3. Generation of Feature Extracted Image

Since half of every pixel in the sensor is covered by the mask, the brightness of *g*(*x*, *y*) is lower than that of *f*(*x*, *y*). Since the brightness of both *g_L_*(*x*, *y*) and *g_R_*(*x*, *y*) is lower than that of *f*(*x*, *y*), their first derivatives are also smaller than the derivative of *f*(*x*, *y*). When the sensitivity of the sensor (ISO) increases, the portion of noise also increases. In additional, a defocused image results in the degradation of edges and other details in the image. In order to obtain a high-frequency component that is robust to noise, the DoG of *g_L_*(*x*, *y*) is performed as [25]:

(2) $$D_{\sigma_{i},\sigma_{j}}\left(x,y\right)=\sum_{u=-k}^{k}\sum_{v=-s}^{s}h_{\sigma_{i}}\left(u,v\right)g_{L}\left(x+u,y+v\right)-\sum_{u=-k}^{k}\sum_{v=-s}^{s}h_{\sigma_{j}}\left(u,v\right)g_{L}\left(x+u,y+v\right)$$

where σ*_i_* represents the scale factor of the Gaussian function, *k* and *s* the horizontal and vertical sizes of the neighborhood and *h*_σ*_i_*_(*x*, *y*) the Gaussian kernel. The normalized DoG is expressed as:

(3) $${\tilde{D}}_{\sigma_{i},\sigma_{j}}\left(x,y\right)=\frac{D_{\sigma_{i},\sigma_{j}}\left(x,y\right)-\min\left\{D_{\sigma_{i},\sigma_{j}}\left(x,y\right)\right\}}{\max\left\{D_{\sigma_{i},\sigma_{j}}\left(x,y\right)\right\}-\min\left\{D_{\sigma_{i},\sigma_{j}}\left(x,y\right)\right\}}\times 0.5$$

If the brightness of low illumination images *g_L_*(*x*, *y*) is increased, the noise component in the normalized DoG is also amplified by the difference operation. In order to extract features that are robust to defocus blur without the noise amplification problem, the proposed method additionally uses the difference between the two differently scaled versions of *g_L_*(*x*, *y*) as:

(4) $$S_{\kappa_{i},\kappa_{j}}\left(x,y\right)=\frac{1}{\kappa_{i}^{2}}\sum_{u=0}^{\kappa_{i}-1}\sum_{v=0}^{\kappa_{i}-1}g_{L}\left(x/\kappa_{i}+u,y/\kappa_{i}+v\right)-\frac{1}{\kappa_{j}^{2}}\sum_{u=0}^{\kappa_{j}-1}\sum_{v=0}^{\kappa_{j}-1}g_{L}\left(x/\kappa_{j}+u,y/\kappa_{j}+v\right)$$

where *κ_i_* and *κ_j_* represent two different scale factors. Since *S*_*κ_i_*, *κ_j_*_ (*x*, *y*) is the difference between two mean filtered images with different filter sizes, it can amplify the edge without amplifying the noise. The normalized version of *S*_*κ_i_*, *κ_j_*_ (*x*, *y*) is expressed as:

(5) $${\tilde{S}}_{\kappa_{i},\kappa_{j}}\left(x,y\right)=\frac{S_{\kappa_{i},\kappa_{j}}\left(x,y\right)-\min\left\{S_{\kappa_{i},\kappa_{j}}\left(x,y\right)\right\}}{\max\left\{S_{\kappa_{i},\kappa_{j}}\left(x,y\right)\right\}-\min\left\{S_{\kappa_{i},\kappa_{j}}\left(x,y\right)\right\}}\times 0.5$$

The finally edge-amplified image is obtained by combining Equations (3) and (5) as:

(6) $$p_{L}\left(x,y\right)={\tilde{D}}_{\sigma_{i},\sigma_{j}}\left(x,y\right)+{\tilde{S}}_{\kappa_{i},\kappa_{j}}\left(x,y\right)$$

where *p_L_*(*x*, *y*) ∈ [0, 1], since both *D̃*_σ_*i*_, σ_*j*__ (*x*, *y*) and *S̃*_*κ_i_*, *κ_j_*_ (*x*, *y*) take values in the range [0, 0.5]. Given *p_L_*(*x*, *y*), the positions of strong features can be obtained, and then, the left feature image *g_L_f*(*x*, *y*) is generated by cropping *g_L_*(*x*, *y*) in the neighborhood of the strongest feature. The right feature image *g_R_f*(*x*, *y*) can be obtained from the same coordinate as *g_L_*(*x*, *y*).

**Figure 6.**
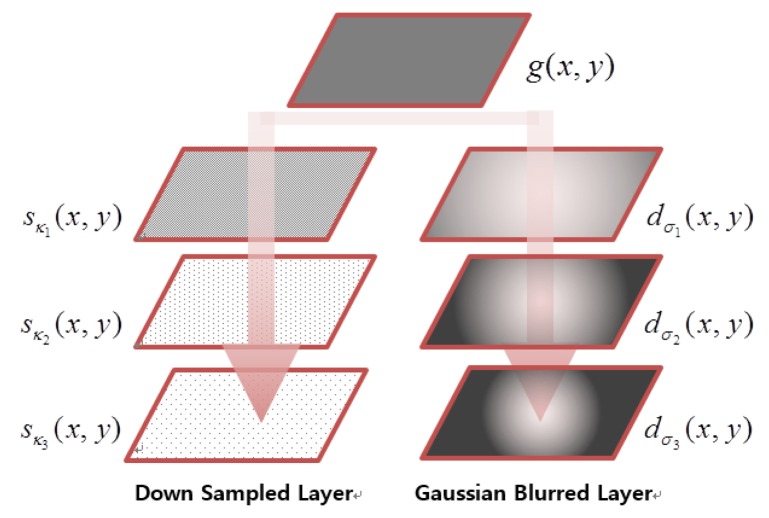
The hierarchical structure of down-sampling and difference of Gaussian (DoG).

To obtain a better outcome, the proposed algorithm performs multi-scale processes by adjusting σ*_i_* and *κ_j_*, as shown in Figure 6, where 2*κ_j_* = *κ_j_*_+1_, and 5σ*_i_* = σ*_i+_*_1_. As a result, the robust feature is extracted from *d*_σ*_i_*_ (*x*, *y*), which has detail edges in the low light and out-of-focus blurred image *g_L_*(*x*, *y*).

### 3.4. Motion Estimation for Moving the Focusing Lens

As shown in Figure 3, the light through the lens reaches the left region of the photodiode when the mask is installed in the right side, and *vice versa*. Thus, two phase images with a symmetric phase difference are acquired from masks when an input image *g*(*x*, *y*) is first sampled at AF points. Next, the high frequency components of images are shifted in the horizontal direction. However, there is no phase difference in the vertical direction, because the height of a mask is equal to that of the photodiode.

**Figure 7.**
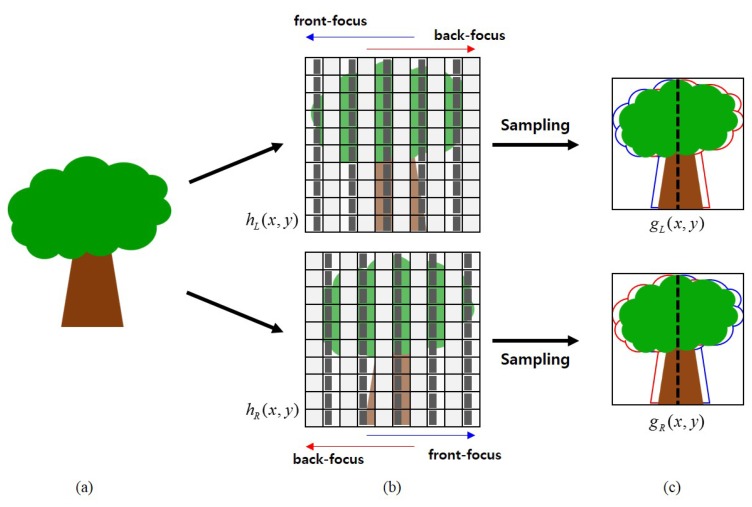
Phase difference generation process: **(a)** an object in the real world; **(b)** left (top) and right (bottom) phase sensor configuration with the appropriate masking; and **(c)** the acquired left (top) and right (bottom) phase images.

Figure 7 illustrates the process of phase difference generation. The light reflected by an object in the real world shown in Figure 7a comes into the camera. As shown at the top of Figure 7b, the left-phase image *g_L_*(*x*, *y*) is generated by the right half masking of photodiodes in the odd columns of the sensor within *h_L_*(*x*, *y*), as shown at the top of Figure 7c. The right-phase image is also generated in the same manner as shown at the bottom of Figure 7b,c. Based on the concept of the sensor-based phase difference auto-focusing system shown in Figure 2, the two images shown in Figure 7c have different locations of the object depending on the distance from the camera. Since the size of black masks is one half of each photodiode, the phase difference is generated with sub-pixel precision.

The proposed system uses hierarchical two-dimensional phase correlation to estimate a sub-pixel motion vector between two phase images. The cross correlation is first estimated from the power spectra of two images using Fourier transform as:

(7) $$E_{PC}\left(\mu ,\nu \right)=\frac{ℑ\left\{g_{Rf}\left(x,y\right)\right\}\times ℑ*\left\{g_{Lf}\left(x,y\right)\right\}}{\left|ℑ\left\{g_{Rf}\left(x,y\right)\right\}\times ℑ*\left\{g_{Lf}\left(x,y\right)\right\}\right|}$$

where (μ, ν) represents the two-dimensional spatial frequency variables, ℑ{·} the two-dimensional Fourier transform operation and ℑ*{·} the complex conjugate of the Fourier transform. Next, the proposed method performs the inverse Fourier transform of *E_PC_*(μ, ν) to estimate the motion vector by selecting the maximum value as:

(8) $$\left(\Delta x,\Delta y\right)=\max\left[{ℑ}^{-1}\left\{E_{PC}\left(\mu ,\nu \right)\right\}\right]$$

where (Δ*x*, Δ*y*) represents the phase difference between *g_Lf_*(*x*, *y*) and *g_Rf_*(*x*, *y*). For example, if there is no phase difference between *g_Lf_*(*x*, *y*) and *g_Rf_*(*x*, *y*), we have that (Δ*x*, Δ*y*) = (0, 0).

**Figure 8.**
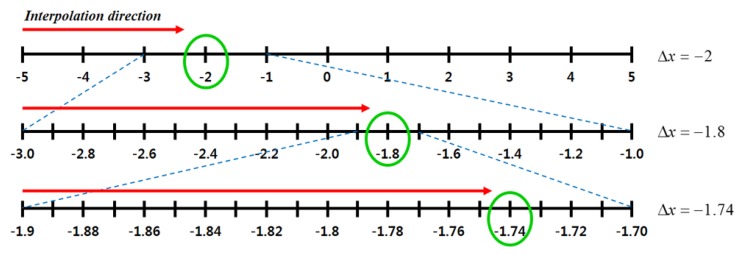
The proposed hierarchical phase correlation method.

The phase correlation method is robust to the degradation of the Fourier magnitude and less affected by noise than other registration methods [26]. However, the original version of the phase correlation method cannot be used, since it cannot provide sub-pixel resolution. Therefore, the proposed system modifies the phase correlation method using hierarchical interpolation to estimate the motion vector in the sub-pixel precision. The proposed method takes only Δ*x*, because the masks cover the pixels in the horizontal direction, as shown in Figure 3. Figure 8 shows the proposed hierarchical interpolation method. *g_Lf_*(*x*, *y*) is first interpolated to fill in every integer pixel in the range of [−5, 5]. Next, the integer motion vector (Δ*x*, Δ*y*) is estimated using the phase correlation methods, yielding *g_Lf_*(*x* − Δ*x*, *y* − Δ*y*), which is aligned to *g_Rf_*(*x*, *y*) with the integer precision. The same process repeats twice with precisions of 0.1 and 0.01.

## 4. Experimental Results

The proposed auto-focusing system was simulated on a personal computer with a 3.70-GHz Intel® Core™ i7 CPU and 16 GByte of memory. Test images were acquired by a digital camera equipped with an APS-Ctype CMOS image sensor with 20 million pixels and a 50-mm F1.8 lens.

**Figure 9.**
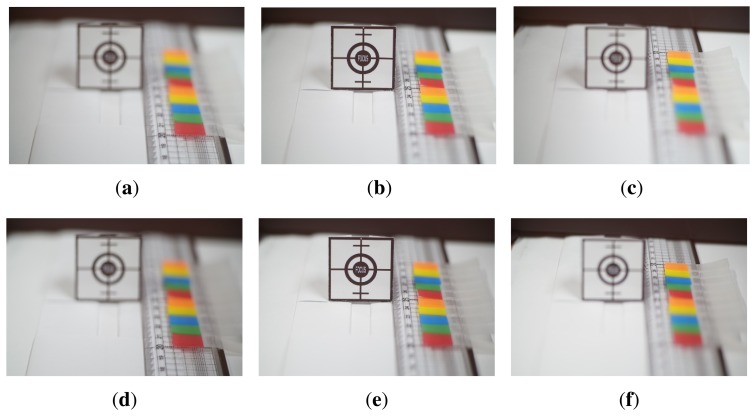
The set of differently-focused test images: **(a)** left-phase, front-focus image; **(b)** left-phase, in-focus image; **(c)** left-phase, far-focus image; **(d)** right-phase, front-focus image; **(e)** right-phase, in-focus image; end **(f)** right-phase, far-focus image.

The proposed system was tested with a sufficient amount of illumination of 400 lux. Figure 9 shows a set of test images of a size of 1610 × 1080 with different focus distances. Figure 9a–c shows left-phase images focused at 20, 25 and 30 cm, respectively. Figure 9d–f shows right-phase images focused at 20, 25 and 30 cm, respectively. In addition to the test images shown in Figure 9, we also acquired eleven pairs of left- and right-phase images at the focus range of 20 to 30 cm.

**Figure 10.**
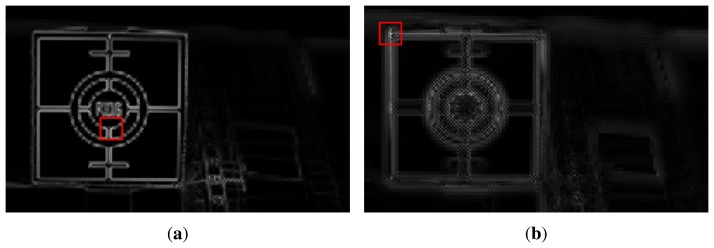
Results of feature extraction in the cropped region of Figure 9: **(a)** the feature of the in-focus image shown in Figure 9b,c and **(b)** that of the front-focus image shown in Figure 9a.

The results of feature extraction in the cropped region of size a of 1000 × 600 centered at (750,500) are shown in Figure 10. The red box in Figure 10a,b, respectively, shows 64 × 64 square regions containing the strongest features in Figure 9a,c. Since Figure 9b is an in-focus image, Figure 10a has stronger features than other images. In the same manner, since Figure 9a is a front-focus image, Figure 10b has weaker features than Figure 10a.

**Figure 11.**
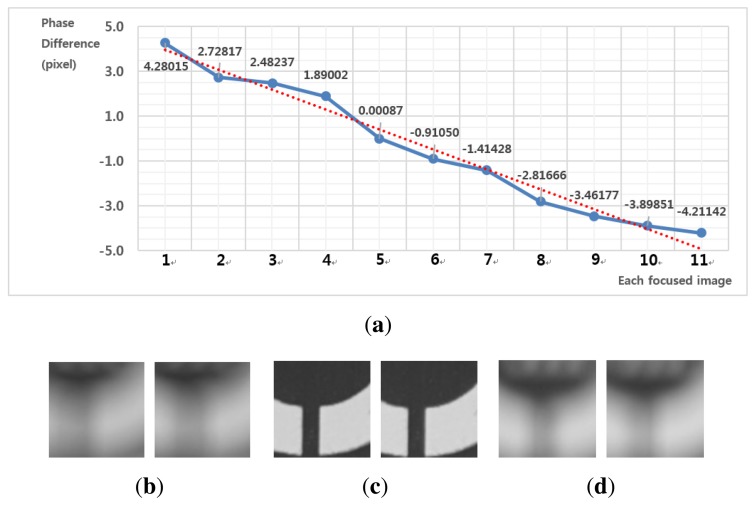
The results of motion estimation for different phases: **(a)** estimated motion vectors *versus* the phase difference; **(b)** pair of left- and right-phase images at the front-focus position (Image 1) with a phase difference of 4.28015; **(c)** at the in-focus position (Image 5) with a phase difference of −0.91050; and **(d)** at the far-focus position (Image 11) with a phase difference of −4.21142.

Figure 11a shows results of motion estimation for different phases. Figure 11b–d shows pairs of left- and right-phase images at front-, in- and far-focus positions, respectively. As shown in Figure 11a, the experimental curve follows the dotted line, which demonstrates that the proposed method can accurately represent the linear relationship between the phase difference and the motion vector. As shown in Figure 11b,d, motion vectors are robustly estimated from pairs of significantly blurred images.

**Figure 12.**
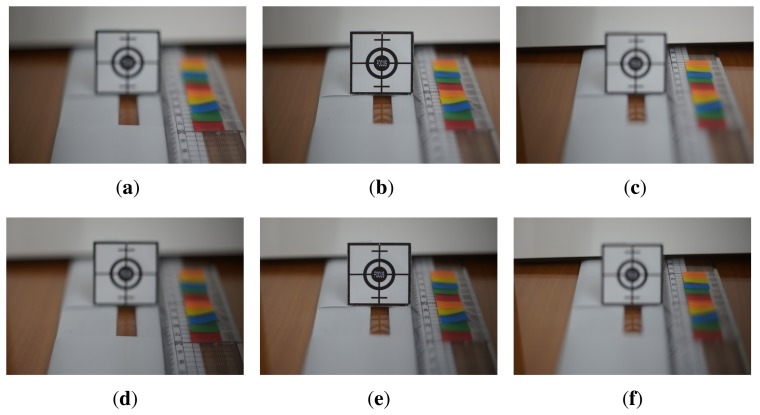
The set of differently-focused test images with 20 lux: **(a)** left-phase, front-focus image; **(b)** left-phase, in-focus image; **(c)** left-phase, far-focus image; **(d)** right-phase, front-focus image; **(e)** right-phase, in-focus image; and **(f)** right-phase, far-focus image.

For the test of the low light environment, the illumination was changed down to 20 lux, as shown in Figure 12 for a size of 2448 × 1624. Figure 12a–c shows the left-phase images focused at 20, 25 and 30 cm, whereas Figure 12d–f shows the right-phase images focused at the same focus distances as shown in Figure 12a–c.

**Figure 13.**
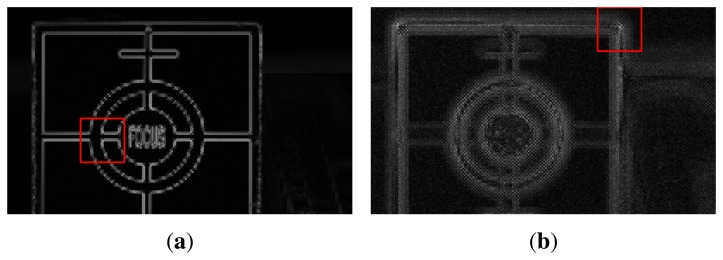
Results of feature extraction in the cropped region of Figure 12: **(a)** the feature of the in-focus image shown in Figure 12b,d and **(b)** that of the front-focus image shown in Figure 12a.

Figure 13 shows the results of feature extraction in the cropped region of a size of 128 × 128 centered at (276, 386). Figure 13 shows the strongest feature-extracted versions of Figure 12a,b. Figure 13a, that is the in-focus image, has the strongest features without noise. Although Figure 13b is a feature extraction result of the front-focus image with out-of-focus blur, meaningful features are successfully extracted.

**Figure 14.**
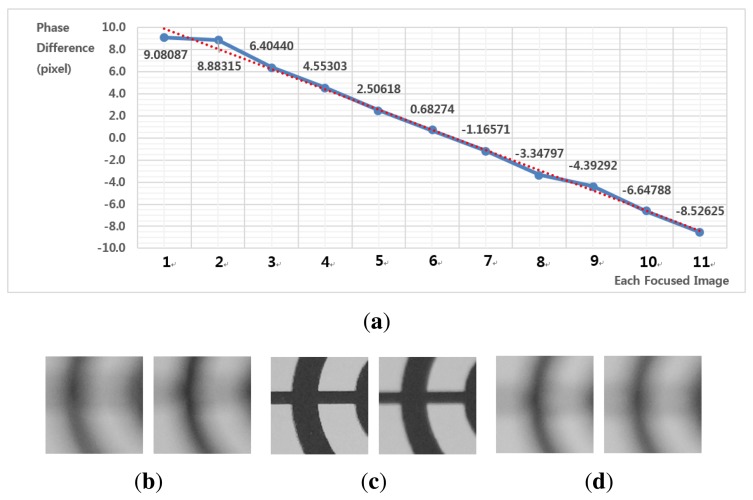
Results of motion estimation for different phases: **(a)** estimated motion vectors *versus* the phase difference; **(b)** pair of left- and right-phase images at the front-focus position (Image 1) with a phase difference of 9.08087; **(c)** at the in-focus position (Image 6) with a phase difference of −0.68274; and **(d)** at the far-focus position (Image 11) with a phase difference of −8.52620.

Figure 14a shows estimated motion acquisition results from the image set shown in Figure 13. Figure 14b–d shows pairs of left and right-phase images at the front-, in- and far-focus position. As shown in Figure 14a, the proposed method can preserve the linear relationship in a low light environment.

**Figure 15.**
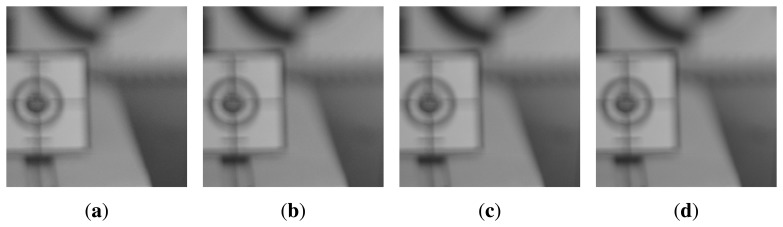
The set of front-focused left-phase images with different illumination : **(a)** the 5 lux image with ISO-3200; **(b)** the 40 lux image with ISO-2000; **(c)** the 70 lux image with ISO-1000; and **(d)** the 100 lux image with ISO-800.

To verify the powerful method in the defocus condition under low illumination, the final experiment was performed with the illumination level changed from 5 to 100 in 5 lux increments . Figure 15 shows a set of front-focused left-phase images of a size of 1200 × 1200 with each 10, 40, 70 and 100 lux.

**Figure 16.**
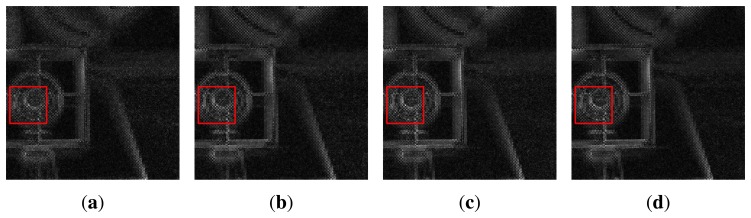
Results of the feature extraction of the left-phase images of Figure 15: **(a)** the 5 lux feature image with ISO-3200; **(b)** the 40 lux feature image with ISO-2000; **(c)** the 70 lux feature image with ISO-1000; and **(d)** the 100 lux feature image with ISO-800.

Figure 16 represents the results of the feature extraction of Figure 15 of a size of 600 × 600 with 128 × 128 square regions. The proposed method can extract the strongest feature, although Figure 16 has different illumination levels and sensor sensitivities in each subfigure.

**Figure 17.**
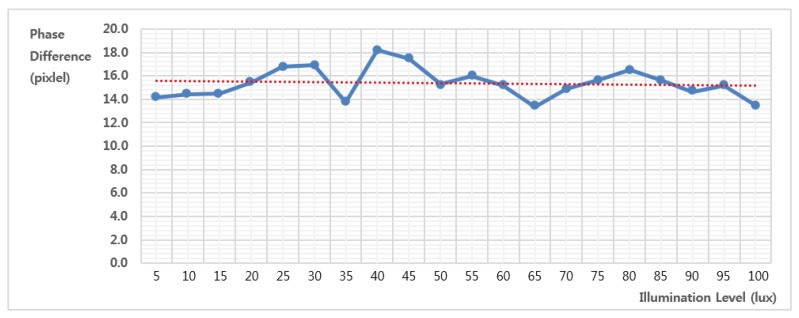
Results of motion estimation for different illumination levels (the unit of the vertical axis is in pixels, and that of the horizontal axis is in lux).

Figure 17 shows the motion estimation results from the image set of Figure 15. As shown in the red dotted line, the proposed system can estimate the motion value regardless of the illumination level, although each image has a small error, as shown by the blue solid line.

## 5. Conclusions

In this paper, a novel sensor-based phase difference auto-focusing system is presented to realize a robust, accurate hybrid AF system. The proposed system is robust to optical error in estimating the phase difference, and it can accurately detect strong features in a low light environment. An additional contribution of this work is providing the displacement of lens movement using only photodiodes in the main image sensor instead of using an additional auto-focusing sensor, as in existing PDAF systems. The experimental results demonstrate that the proposed system can accurately estimate the displacement in the entire range of the focus position. As a result, this system can acquire an in-focus image regardless of the optical path or external conditions, such as low light environment. In the aspect of implementation efficiency, the proposed auto-focusing system does not need an additional sensor to detect the phase difference, but uses the main image sensor by partially masking the selected photodiodes. For this reason, the proposed system serves as both algorithmic and systematic frameworks of hybrid auto-focusing systems. However, the proposed system loses a portion of illumination, since the photodiodes are partially masked. The problem of losing illumination should be addressed in future research based on either sensor technology or digital signal processing algorithms. Furthermore, phase correlation cannot exactly estimate the motion vector in repetitive pattern objects. Therefore, more elaborate motion estimation needs to be researched in the future.
